# Assessment of *Megadenus holothuricola* Rosén, 1910 (Eulimidae), an endoparasite of *Holothuria mexicana* Ludwig, 1875 (Holothuriidae) in the southern Gulf of Mexico and the description a new species

**DOI:** 10.3897/zookeys.1016.55071

**Published:** 2021-02-11

**Authors:** Norma Emilia González-Vallejo, Stephanie Amador-Carrillo

**Affiliations:** 1 El Colegio de la Frontera Sur, Depto. Sistemática y Ecología Acuática, Av. Centenario km. 5.5 Chetumal, Quintana Roo, CP. 77014, Mexico El Colegio de la Frontera Sur Chetumal Mexico

**Keywords:** Campeche, Eulimidae, gastropods, holothurians fishery, new species, Palmyra Atoll, symbiosis

## Abstract

As part of a study on holothurians from the southern Gulf of Mexico, some *Holothuria mexicana* Ludwig, 1875 were obtained for gut analysis. In two of them, a couple of eulimids were located inside the main tube of the respiratory tree. They were identified as *Megadenus holothuricola* Rosén, 1910, described from the Bahamas Islands, based on five specimens attached to the respiratory tree of *H. mexicana*. The original description was brief with few details, the type material is lost, and the species has not been found again. In this contribution, this species is confirmed for Campeche Bay, Mexico. The adult shell is globular to conical, transparent, thin, and fragile. *Megadenus smithi***sp. nov.** from Palmyra Atoll, Central Pacific is described based on adult specimens. It differs from its congeneric species in its more robust shell, the pseudopallium does not cover the shell, and its short and contracted proboscis forms a thick disc. Further research on these eulimid parasites is now complicated in the southern Gulf of Mexico because of the holothurian population collapse due to over-exploitation of the fishery.

## Introduction

Eulimidae form a large group of parasitic snails infesting sea urchins, holothurians, starfish, and feather stars. Genera vary in their physical characteristics and lifestyle (Heller 2015). Some species live permanently attached to their echinoderm host; others attack their prey only temporarily and fall off if disturbed. Therefore, they are usually collected apart from their host, which thus may remain unknown (Warén 1984). However, many eulimid species are found to live on the body surface like ectoparasites, or as endoparasites especially in holothurians, accomplished by a series of morphological and anatomical changes (Nekhaev 2016; González-Vallejo 2018; Souza et al. 2018).

Our knowledge of marine parasites is fragmentary. This observation, especially in the case of invertebrate hosts, is probably caused by their low occurrence rates (Boxshall 2020). In Mexico, there are no records so far for eulimid endoparasites in holothurians; one ectoparasitic species was reported as *Melanella intermedia* (Cantraine, 1835), originally described from the Mediterranean Sea, in the skin of Holothuria (Halodeima) grisea Selenka, 1867 and *Holothuria glaberrima* Selenka, 1867. Both holothurians were reported from Veracruz in the Gulf of Mexico (Caso 1968, 1971).

The fishery of holothurians has increased in Campeche Bay during the last eight years. As part of a study of their feeding habits, some holothurians were obtained. After examining the internal organs, several endoparasites were located attached to or immersed in the main tube of the respiratory tree of Holothuria (Halodeima) mexicana Ludwig, 1875. One of the endoparasitic species was identified as *Megadenus holothuricola* Rosén, 1910. This eulimid was found attached to the respiratory tree of *H. mexicana*. The type locality of this species is the Bahamas Islands; the type series contained five specimens, and the original shell description was brief with few details only. The type material is lost, and the species has not been found again (Warén 1984). The genus *Megadenus* Rosén, 1910 includes five species found all around the world, and all of them live in an endoparasitic relationship with holothurian species. The type species *M. holothuricola* was reported from *H. mexicana*; *M. voeltzkowi* Shepman & Nierstrasz, 1913 from H. (Holothuria) pardalis? Selenka, 1867 from Zanzibar, East Africa; *M. cantharelloides* Humphreys & Lützen, 1972 from *Stichopus chloronotus* Brandt, 1835 in Picard Island, Aldabra, Indian Ocean; *M. oneirophantae* Bouchet & Lützen, 1980 from *Oneirophanta mutabilis* Thèel, 1879 off W bay of Biscay, France and *M. atrae* Takano, Warén & Kano, 2017 from H. (Halodeima) atra Jaeger, 1833 from Japan, New Caledonia, India and Australia (see Table 1).

**Table 1.** Comparison of the main character states separating the species of *Megadenus*. The information provided is based on literature (Schepman and Nierstrasz 1913; Humphreys and Lützen 1972; Bouchet and Lützen 1980; Takano et al. 2017). For *M. holothuricola* and *M. smithi* sp. nov. data obtained from specimens.

Species	Shell/size/sex	Pseudopallium	Proboscis	Host/attach site	Geographical distribution & depth
*M. holothuricola* Rosén, 1910	Globose to conical, thin, transparent, fine striae, 4.0–5.5 mm length × 2.0–1.5 mm width	Bowl-shaped with pleated margin	Funnel-shaped, white, flexible, rough cuticular surface; not retractable	*Holothuria mexicana* respiratory tree	Bahamas, Campeche Bay, shallow water
*M. voeltzkowi* Shepman & Nierstrasz, 1913	Ovoid transparent to whitish, slightly smooth 6.5 × 4 mm	Arises from folds of foot	Suction disc form, contracted and wrinkled	*Holothuria pardalis*? esophageal region	Zanzibar East Africa, shallow water?
*M. cantharelloides* Humphreys & Lützen, 1972	Cyrtoconoid and globose, transparent ♀ 5.7 × 4.5 mm	Bowl-shaped fold of tissue, more extensive in male than female	Chanterelle-shaped, darkened, contracted	*Stichopus chloronotus* intestine	Picard Island, Aldabra, India, shallow water
*M. oneirophantae* Bouchet & Lützen, 1980	Globose, vitreous ♀ 9.3 × 7.5 mm, ♂ 3.0 × 1.6 mm	Base partly covered by foot and its folds	Long, thin	*Oneirophanta mutabilis* within galls in intestine	Bay of Biscay, 4796 m abyssal depth
*M. atrae* Takano,Warén & Kano, 2017	Globose, pyriform to biconical, transparent to whitish ♀ 5.2 mm; ♂ 2.0–1.5 mm	Cauldron-shaped	Long, thickened at the middle into a collar-like	*Holothuria atra*, cloacal chamber and respiratory tree	Japan, New Caledonia, India Australia, tidal waters
*M. smithi* sp. nov.	Globose to biconical, transparent,finally axial striae ♀6.5 mm L ♂ 4.0 mm	In folds of foot and cauldron-shaped	Suction disc form, slightly dark colored retractable	*Holothuria atra* cloacal chamber	Palmyra Atoll, shallow water

**Figure 1.**
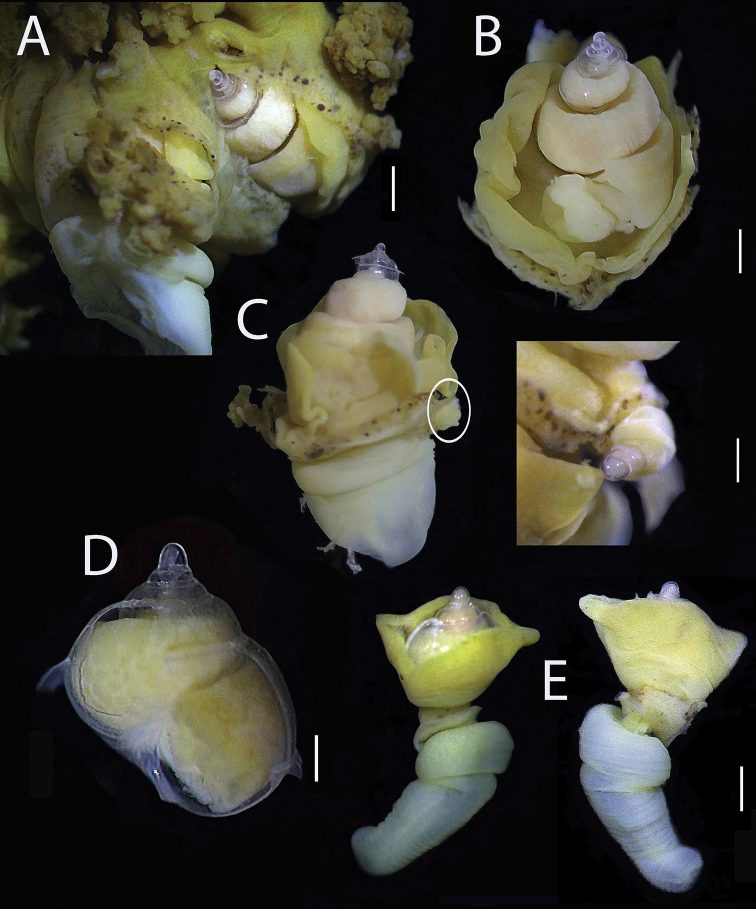
*Megadenus holothuricola* Rosén, 1910 **A** two adults between filaments of the respiratory tree **B, C** detached adult shells, SL = 5.5 mm and 4.5 mm; right side juvenile attached SL = 1.0 mm **D** broken adult shell SL = 2.5 mm **E** subadult SL = 2.0 mm with everted proboscis L = 2.5 mm. Scale bars: 1.6 mm (**A**); 1.8 mm (**B**); 1.4 mm (**C**); 0.2 mm (**D**); 1.1 mm (**E**).

**Figure 2.**
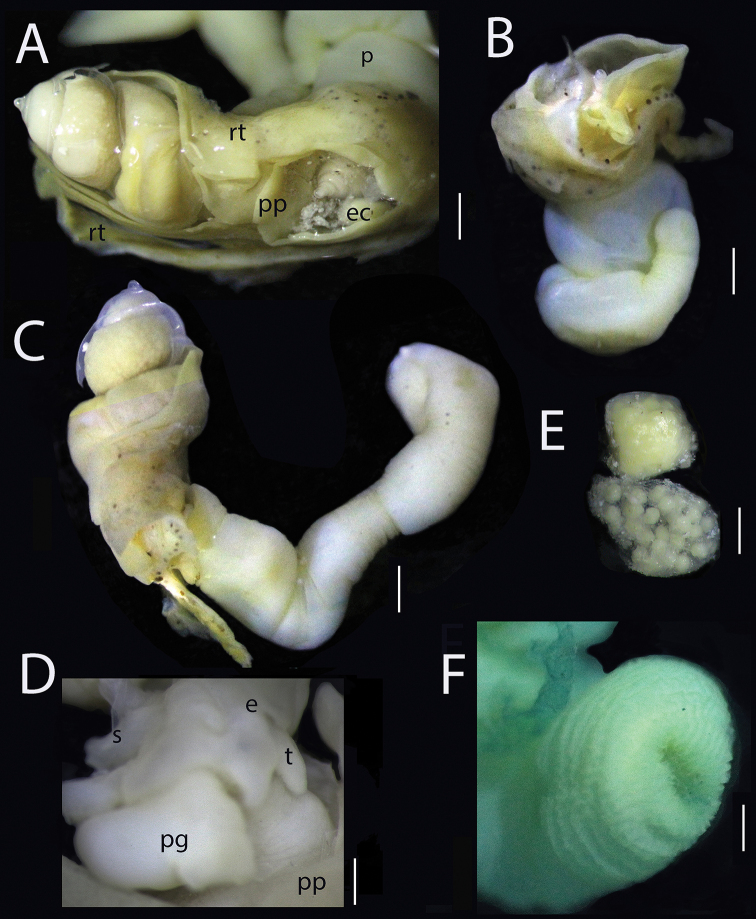
*Megadenus holothuricola* Rosén, 1910 **A** adults immersed in the main tube of the respiratory tree **B, C** two adults showing the mottled skin of the respiratory tree and the pseudopallium covering shell; proboscis slightly rolled, and another adult stretched to the maximum **D** cephalic view, 40× **E** two egg capsules, L = 2 mm **F** view of the distal part of an adult proboscis. Scale bars: 1.2 mm (**A**); 2.3 mm (**B**); 1.65 mm (**C**); 0.8 mm (**D**); 0.6 mm (**E**); 0.5 mm (**F**). Abbreviations: e: eyes; eg: egg capsules; p: proboscis; pg: pedal gland; pp: pseudopallium; rt: respiratory tree; s: shell; t: tentacle.

**Figure 3.**
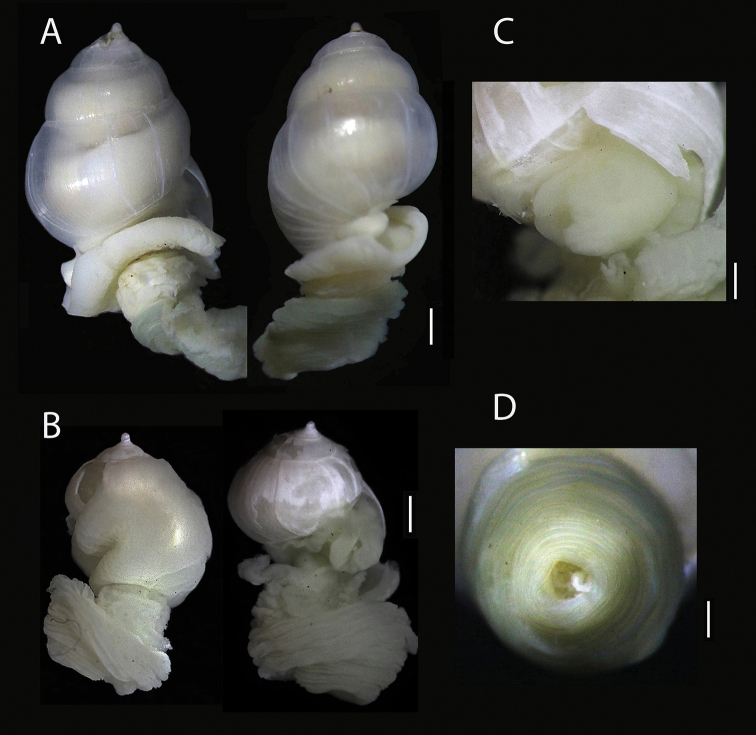
*Megadenus smithi* sp. nov. **A** holotype female in apertural and posterior views (ECOSUR-0234) SL = 6.5 mm × 4.0 mm wide **B** paratype male in apertural and lateral view, showing the tentacles SL = 4.0 mm. × 3.5 mm wide **C** pedal gland view **D** distal open proboscis view. Scale bars: 1.2 mm (**A**); 1.0 mm (**B**); 0.6 mm (**C**); 0.5 mm (**D**).

Rosén (1910) supplied a brief description of the shell of *M. holothuricola* as “conical, shiny, finely cross-striae, fragile, without an umbilicus, without an operculum, with six spiral turns, the first two very small”. He mainly focused on describing the histology of the species (pls 1–4, figs 1–16) comparing the morphological differences of *M. holothuricola* with species from other genera such as *Stilifer* Broderip, 1832, *Mucronalia* Adams, 1860, *Pelseeneria* Koehler & Vaney, 1908, and *Turtonia* Rosén, 1910. Thus, the species’ shell features remain ill-defined, which makes a detailed re-description necessary. In this work, we assess the endoparasitic relationship of *M. holothuricola* with *H. mexicana* in the Bay of Campeche, which also represents the first finding after the original description and the first record for this species from the Gulf of Mexico. Details of the shell and its variation are described, and information on the shells of sub-adults, juveniles, and larvae are provided, as well as comments on morphological features such as proboscis, mantle color, and egg capsules and supported by illustrations. Additionally, a new species is described from Palmyra Atoll, Central Pacific.

## Materials and methods

One hundred holothurians were collected and donated by fisherman for gut-analyses and molecular studies. Holothurians were collected at 5 m depth in mixed sea grass meadows and sandy areas off the coast of Lerma (19°49.31'N, 90°36.51'W), Campeche Bay, Mexico, on 4 May 2012. Two species of holothurians were identified, *H. mexicana* and *H. floridana*; the identification was confirmed by molecular studies and the analysis of calcareous ossicles (following Amador-Carrillo 2014). The holothurians were all adults, 12–22 cm long, and were relaxed in freshwater to avoid evisceration. The holothurians were injected with a 4% formaldehyde solution in sea water and preserved in 70% ethanol. In the laboratory, holothurians were dissected using a sagittal cut, the visceral mass was removed, and placed in individual containers. Three specimens identified as *Megadenus* sp. were collected from Palmyra Atoll, Central Pacific by J. Smith, on 11 September 2011 attached to the cloaca of *H. atra* Jaeger, 1833, were studied and are described as a new species.

The Eulimidae were photographed with a stereomicroscope and a Cannon EOS T6i camera, mounted with an adapter into the ocular tube. Measurements were made with a micro ruler (mm). Digital plates were assembled with Adobe Photoshop CC. Holothurians and eulimids were deposited in the collections of El Colegio de la Frontera Sur (**ECOSUR**) Unidad Chetumal, Mexico.

## Results

The endoparasite *M. holothuricola* was found in only two holothurians. In the first one, there were two adult specimens and one juvenile; all were immersed in the skin of the main tube of the respiratory tree (Fig. 1A). The second holothurian had two adult specimens and one juvenile attached in the same way as the previous specimen, but additionally there were four probosces, without shells or specimens (Fig. 2A). In total, there were three adults and three juveniles or sub-adults. The prevalence of the endoparasites in the host was 2% of the total holothurians examined.

### Taxonomy

#### Class Gastropoda Cuvier, 1795

**Order Littorinimorpha Golikov & Starobogatov, 1975**

**Familia Eulimidae Philippi, 1853**

##### Genus Megadenus

Taxon classificationAnimaliaLittorinimorphaEulimidae

Rosén, 1910

406FF038-AE8A-5ACD-8F5C-1A7A658D98D9

###### Type species.

*Megadenus holothuricola* Rosén, 1910:18 by monotypy.

###### Diagnosis.

Shell conical to globular, translucent, colorless, sculpture of fine axial striae. Pseudopallium covers the shell or not; proboscis large or short forming a thick contracted disc; large pedal gland (Rosén 1910; Humphreys and Lützen 1972).

###### Remarks.

*Megadenus* species are found in shallow waters in tropical or subtropical regions, especially as endoparasites of Aspidochirotida (Echinodermata, Holothuroidea); usually they live in the cloacal chamber or attached to the respiratory tree. *Megadenus oneirophantae* Bouchet & Lützen, 1980 is the only abyssal species (4796 m). Warén (1984) defined *Megadenus* as “shell fragile, globular, and at least lower part is covered by a big pseudopallium, has separate sexes, with smaller males and the animals live in the cloaca of holothurians.” He also indicated there was no type material.

##### Megadenus holothuricola

Taxon classificationAnimaliaLittorinimorphaEulimidae

Rosén, 1910

F912BFF9-3894-52F5-84A0-096AFAE50E97

Figs 1A–E , 2A–F

Megadenus holothuricola Rosén, 1910:18–49 pl. 1 figs 1, 2 (type locality: Bahamas Islands; Type material lost).

###### Specimens examined.

four adult specimens ECOSUR-1386, two juveniles ECOSUR-1387 from Lerma, Campeche Bay, Gulf of Mexico.

###### Distribution.

Bahamas and Lerma, Campeche, Mexico.

###### Description.

***Shell*** conical to globose, translucent, thin, fragile, sculpture consists of fine striations and growth scars from earlier positions of outer lip, two or three in random position per whorl. Adult size 4.0–5.5 mm long and 2.5–3.5 mm wide range (N = 6) (Fig. 1B, C); teleoconch 4–6 very convex whorls, body whorl comprising more than 50% of the total shell length, posterior whorls reduced in size, sutures well defined aperture broad, inner lip broken, rounded basally; outer lip thin, slightly convex in lateral view, no operculum or periostracum; protoconch mucronate, 1½ whorls, translucent, rounded apex; sub-adults shell globose, low spiral, 2½ whorls, 1.0–2.0 mm long, 1.0 mm wide; body whorl half as long as body and more globose than in adults.

***Body*** fully retractable into shell, mantle light cream-colored, pseudopallium whitish to light yellow in color; short and rounded tentacles, eyes round, black at the base of each tentacle (Fig. 2D); foot reduced with a huge marginal gland of unknown function in dorsal position. The anterior region has two structures arising next to the mantle, the pseudopallium surrounding and partially covering the shell forming an extensive fold with pleated edges, and the proboscis. Epithelium fused with the holothurian respiratory tree; proboscis only partially fused with a part of the pseudopallium and foot, protruding from pseudopallium folds; in juveniles, pseudopallium covering lateral areas, leaving the apical teleoconch exposed; in 1 mm long juveniles, a small fold distinguishable (Figs 1E, 2C).

***Proboscis*** funnel-shaped, forming a flexible, semi-transparent or white non-retractable tube (Fig. 2C); inside is a series of long and circular muscular packs giving it an elastic morphology and a rough outer cuticular surface; on the distal part is a series of papillae, allowing it to adhere like a suction cup to the epidermis of the host’s respiratory tree (Fig. 2F). The proboscis size varies with total shell size: in adults, it is 3 × longer than the shell, 7.0 mm stretched to the maximum, in sub-adults or juveniles the proboscis is smaller and smoother (Fig. 2B, C).

###### Variations.

The larval shell shows some intraspecific variations in adults. It is glassy, low, and rounded to mucronate. This seems to be related to the protection of the pseudopallium when it is immersed inside the respiratory tube. The juvenile develops the pseudopallium relatively early, when it has 1.5 whorls, and protects the shell from this stage onwards; at the same time, it is attached to an area of the host’s skin respiratory tree (Fig. 1C, E).

###### Remarks.

Inside the posterior whorls of the shell, all adults and subadults presented pink oocytes or germinal cells. Two oval-shaped egg capsules were found situated between the shell and the pseudopallial folds, in one specimen (Fig. 2A, E). Each capsule was transparent, 1.5 mm long × 1.0 mm wide, with 25–30 embryos in each. A juvenile was found strongly attached to the skin layer of the respiratory tube of the host, in the anterior area close to an adult eulimid (Fig. 1C). Shells were difficult to preserve complete due to their fragility.

Rosén (1910) illustrated an adult and its coiled proboscis. His figures 1 and 2, show an adult with the pseudopallium at the top, and the proboscis of another adult separated from the respiratory tree tube. Our results are similar. The shape and sculpture of our shells correspond to Rosén’s original description; that is, a conical shape in pre-adults to globose shell, wider in the body whorl, two posterior smaller whorls, and with fine axial striations and continuous incremental growth lines in adults. The shells of *M. catharelloides* and *M. voeltzkowi* were described and illustrated, but subsequently destroyed to make histological cuts. In *M. holothuricola* there are no shells preserved as type material, hindering a better comparison, as noted by Warén (1984). However, shell shape of the pre-adults or juveniles of *M. holothuricola* resemble adults of *Monogamus minibulla* Olsson & McGinty, 1958 or juvenile specimens of *Pelseeneria* (Koehler & Vaney, 1908). Therefore, the morphological comparison of shells can be misleading, although these genera parasitize sea urchins (González-Vallejo 2008; Delongeville et al. 2011).

##### Megadenus smithi sp. nov.

Taxon classificationAnimaliaLittorinimorphaEulimidae

E422A224-21B4-5007-BB16-5FE591C3ED61

http://zoobank.org/B1F27E92-096F-4D99-8697-EBA5F1CDD2B2

Fig. 3A–D

###### Type material.

***Holotype*** (ECOSUR-0234) female; paratypes (ECOSUR-0235) two males, one juvenile from the type locality found attached in the cloacal chamber of Holothuria (Halodeima) atra Jaeger, 1833 collected on 11 September 2011 by J Smith.

###### Type locality.

Palmyra Atoll, Central Pacific Ocean, shallow water in reef lagoon.

###### Etymology.

This species is being named after the collector Mr. J. Smith, as a means of recognition, for his sampling efforts.

###### Distribution.

Known only from the type locality.

###### Diagnosis.

Female shell globose to conical, transparent, fragile, glassy, colorless, fine incremental axial stria; apex mucronate glassy white not tilt; proboscis slightly dark; large pedal gland.

###### Description.

***Shell*** globose, translucent, thin, fragile, colorless, smooth sculpture, with fine axial striae, several growth scars of earlier outer lip positions are irregularly spaced, suture impressed. Adult shell 6.5 mm long; 4.0 mm wide; body whorl 4.5 mm long (holotype Fig. 3A). Teleoconch 5.5 convex whorls, three flat, small post-larval whorls, the penultimate whorl 1.0 mm. Aperture broad, inner lip smooth, concave at base, outer lip simple, slightly curved in most protruding part. Protoconch mucronate, two whorls, glassy white, no operculum or periostracum; sexually dimorphic, male with a penis behind the eye, female is 30% larger than male (Fig. 3B).

***Soft tissues*** pale cream with reddish pigmentation, seen through transparent shell. Pseudopallium cauldron-shaped in sub-adults arising from the aperture and covering large part of shell, or short mantle folds not covering female shells, smooth. When skin was removed, a thick, rounded pedal gland was visible in the aperture (Fig. 3C). Head with a pair of thick long tentacles and black eyes; penis large, placed behind right eye in males. Proboscis slightly dark colored, retractile, separate from the pseudopallium by a deep constriction. Proboscis skin slightly contracted after fixation, forming a thick disc with the mouth distally (Fig. 3D), almost as long as whole specimen. No egg capsules or juveniles were found.

###### Remarks.

The new species shares several shell and morphological traits with the other five congeneric species. A comparison of the most important structures, almost all based on the literature, is provided in Table 1. *Megadenus smithi* sp. nov. resembles *M. voeltzkowi* because both have a tall shell (6.5 mm), a swollen and more convex penultimate whorl than *M. holothuricola* and *M. atrae*. The protoconch in *M. holothuricola* is transparent, 1.5 whorls, and is slightly wider than high, while in *M. smithi* sp. nov. it is two whorls higher than wide much like in *M. cantharelloides*, *M. voeltzkowi*, and *M. atrae*. However, the latter has a slightly tilted protoconch axis. *Megadenus oneirophantae* has a long thin proboscis, the pseudopallium is missing, and the species exhibits a marked sexual dimorphism with a dwarf male.

## Discussion

*Megadenus holothuricola* Rosén, 1910 was described from the Bahamas Islands, Caribbean, more than one hundred years ago. The low occurrence of finding this kind of endoparasites, 2% as in this study, had already been reported elsewhere: Jones and James (1969) reviewed 1,300 specimens of *H. atra* from the Gulf of Mannar, India, and found only eight holothurians with eulimids (0.6%), Humphreys and Lützen (1972) reviewed one holothurian containing only two eulimids, while Takano et al. (2017) reviewed 3,848 specimens of *H. atra*, and only 1.7% with the associated eulimid endoparasite.

Rosén (1910) illustrated a histological section of the proboscis of *M. holothuricola*, but, following Humphreys and Lützen (1976), he used confusing terminology, for example, a type of cell in the proboscis epithelium which was “neither ciliate nor glandular columnar cells produce cuticular process”, and concluded “only *M. holothuricola* has a cuticular surface in the distal part of the proboscis”. Checking the internal surface of the proboscis, we observed that the cuticular aspect as mentioned by Rosén is caused by inner muscles fibers. Size and retraction abilities of the proboscis differs among the species in the genus. He indicated that “the proboscis is larger in the female”; however, in our study all probosces observed were extended, none was retracted (Fig. 2C). The new species *M. smithi* has a short retracted disc, without papillae in the distal area, without differences in size between male and female specimens (Fig. 3A), but resembles a short, thick, retracted disc as figured for *M. voeltskowi* (Shepman & Nierstrasz, 1913: pl. 1, fig. 1), *M. cantharelloides* Humphreys & Lützen (1972: fig. 2A), and *M. atrae*Goto (2010: fig. 1D, E; Takano et al. 2017: fig. 1D, E). Apparently the differences in the shapes and lengths of the probosces between species are primarily related to the size of the adults or sub-adults, and secondly to the type of host organ it inhabits, which is usually the cloacal chamber or the respiratory tree.

*Holothuria mexicana* is the largest holothurian in the Gulf and Mexican Caribbean Sea, reaching up to 50 cm in length. The hindgut (cloaca) bears highly branched outgrowths that extend anteriorly, the respiratory trees. The function of the pair of respiratory trees is to take oxygen from the water, but at the same time they are excretory organs in combination with the intestine. Nitrogenous waste (ammonia) is carried by coelomocytes to the respiratory trees and released through pumping systems (Brusca and Brusca 1990). According to the above, *M. holothuricola* apparently does not cause any harm to the host and as Rosén (1910) reported, the proboscis floats freely in the body cavity of the holothurian, due to a circular perforation of the respiratory tree tubes that it had made previously. It may obtain food by extracting coelomocytes from the walls of the respiratory tree, or it traps organic microparticles. We observed that this species also lives attached to the outer wall of the intestine during the early stages of development; however, there is no information available on its food nor other activities.

An egg capsule was described and illustrated by Rosén (1910: 57, fig. 1): it is oval with a stalk and was attached to a male shell on a gelatinous mass; he did not mention how many capsules were in a clutch. In *M. holothuricola* from Lerma, two egg capsules each containing only 25–30 embryos were protected between the folds of the pseudopallium and the teleoconch, but we suppose there were more, as in their congeners. The same form of protection was reported for *M. cantharelloides* with 26 egg capsules each containing 85–157 embryos, confirming a continuous production of embryos (Humphreys and Lützen 1972). For *M. atrae*, 11 egg capsules containing ca. 170 embryos in total at different stages of development with small degrees of sexual dimorphism were reported (Takano et al. 2017). The presence of sexual dimorphism was not confirmed in this study. Warén (1980) mentioned five sexual strategies in Eulimidae, and protandric hermaphrodites with environmental sex determination strategy (ESD) seem to be common. Adults of *M. holothuricola* were present in pairs, both shells were the same size, only one had egg capsules, but oocytes and germ cells could be observed in the posterior whorls in both specimens. It has been shown that *M. atra* is dimorphic, lives in pairs, and the sexes are separate with males being smaller than females (Takano et al. 2017), who assumed that the species of *Megadenus* follow the ESD strategy. In *Megadenus smithi* sp. nov. from Palmyra, a penis could be observed in the cephalic dorsal area. The specimen had a smaller shell and thus exhibits sexual dimorphism, which is part of the sexual strategy ESD. However, *M. holothuricola* may follow the simultaneous hermaphrodites strategy (SH) but it is difficult to fully confirm without either detailed growth and reproductive cycle or histological analysis.

Another pending question is how these endoparasites migrate from one to another holothurian. Juveniles were found attached to the pseudopallial wall of an adult specimen and was immersed in the same main respiratory tree tube of the host (Fig. 1C, white circle). This finding indicates a brief larval development and displacement of the juvenile within the same host specimen. For long-distance dispersal, the larvae perhaps use the water exchange currents produced in the cloaca by the holothurian (Humphreys and Lützen 1972), or they could be released during the periodical evisceration events of *Holothuria*. Evisceration may be caused by pollution, or as a defense mechanism. During this phenomenon, the cloaca is expelled as well as parts of the respiratory tree, the digestive system, and the gonads (Barnes 1984). In these cases, the juveniles would have the opportunity to find and colonize another host specimen.

Further research on this eulimid parasites is now compromised in the southern Gulf of Mexico, because the holothurian populations have collapsed due to over exploitation. This illegal practice has been interdicted by a law that prohibits the extraction of holothurians in the area (DOF 2013).

## Supplementary Material

XML Treatment for Megadenus

XML Treatment for Megadenus holothuricola

XML Treatment for Megadenus smithi
